# Neutrophil Oxidative Burst Profile Is Related to a Satisfactory Response to Itraconazole and Clinical Cure in Feline Sporotrichosis

**DOI:** 10.3390/jof10060422

**Published:** 2024-06-14

**Authors:** Luisa Helena Monteiro de Miranda, Marta de Almeida Santiago, Julia Frankenfeld, Erica Guerino dos Reis, Rodrigo Caldas Menezes, Sandro Antonio Pereira, Isabella Dib Ferreira Gremião, Regina Hofmann-Lehmann, Fátima Conceição-Silva

**Affiliations:** 1 Sydney School of Veterinary Science, Faculty of Science, The University of Sydney, Sydney, NSW 2006, Australia; 2 Laboratory of Immunoparasitology, Oswaldo Cruz Institute, Oswaldo Cruz Foundation, Rio de Janeiro 21041-250, Brazil; marta.santiago@ioc.fiocruz.br (M.d.A.S.); fconcei@ioc.fiocruz.br (F.C.-S.); 3 Laboratory of Clinical Research on Dermatozoonoses in Domestic Animals, Evandro Chagas National Institute of Infectious Diseases, Oswaldo Cruz Foundation, Rio de Janeiro 21041-250, Brazil; erica.reis@bio.fiocruz.br (E.G.d.R.); rodrigo.menezes@ini.fiocruz.br (R.C.M.); sandro.pereira@ini.fiocruz.br (S.A.P.); isabella.dib@ini.fiocruz.br (I.D.F.G.); 4 Clinical Laboratory and Center for Clinical Studies, Vetsuisse Facility, University of Zurich, 8057 Zurich, Switzerland; julia.frankenfeld@googlemail.com (J.F.); rhofmann@vetclinics.uzh.ch (R.H.-L.)

**Keywords:** sporotrichosis, cat, *Sporothrix*, neutrophils, oxidative burst, flow cytometry

## Abstract

Despite the central role of cats in the transmission and amplification of *Sporothrix*, studies regarding immune response in feline sporotrichosis are scarce. In cats with sporotrichosis, neutrophil-rich lesions are usually associated to good general condition and lower fungal burden. However, the role of neutrophils in anti-*Sporothrix* immunity has been little explored in cats. Thus, the aim of this study was to evaluate the neutrophil oxidative burst in the blood of cats with sporotrichosis. Cats with sporotrichosis included in the study were treated with itraconazole (ITZ) alone or combined with potassium iodide (KI). The neutrophil oxidative burst was evaluated through a flow-cytometry-based assay using dihydrorhodamine 123 (background) and stimulation with Zymosan and heat-killed *Sporothrix* yeasts. The cure rate was 50.0% in cats under treatment with ITZ monotherapy and 90.9% in cats treated with ITZ + KI (*p* = 0.014), endorsing the combination therapy as an excellent alternative for the treatment of feline sporotrichosis. Higher percentages of *Sporothrix*-stimulated neutrophils were associated with good general condition (*p* = 0.003). Higher percentages of *Sporothrix*- (*p* = 0.05) and Zymosan-activated (*p* = 0.014) neutrophils before and early in the treatment were related to clinical cure in ITZ-treated cats. The correlation between oxidative burst and successful use of KI could not be properly assessed given the low number of failures (*n* = 2) in this treatment group. Nasal mucosa involvement, typically linked to treatment failure, was related to lower percentages of activated neutrophils in the background at the treatment outcome (*p* = 0.02). Our results suggest a beneficial role of neutrophils in feline sporotrichosis and a positive correlation between neutrophil activation and the cure process in ITZ-treated cats.

## 1. Introduction

Sporotrichosis is a mycosis caused by *Sporothrix* species and has emerged in the last few decades as an important zoonotic disease related to scratches and bites from infected cats, especially in Brazil. The advance of the highly pathogenic and cat-transmitted species *S. brasiliensis* from Brazil to other countries in South America as well as the increasing occurrence of zoonotic transmission of *S. schenckii* in Malaysia are concerning and highlight the importance of understanding the disease in cats.

Feline sporotrichosis usually develops as single to multiple ulcerated skin lesions that typically present high fungal burdens, which explains in part cats’ great zoonotic potential for transmission of *Sporothrix* spp. The involvement of nasal mucosa and the presence of respiratory signs are also commonly reported and considered challenging in terms of treatment. Severe disease, including systemic involvement and fatal outcomes, is frequent in cats [1,2] in comparison to other hosts.

The treatment of feline sporotrichosis is primarily with itraconazole (ITZ), which is often effective. However, disease progression, persistence and recurrence of clinical signs are still described in cats treated under this regimen, especially when there is nasal mucosa involvement or respiratory signs. In these cases, the use of the combination therapy of ITZ and potassium iodide (KI) has resulted in increased cure rates [3] and is the protocol recommended by the Brazilian guideline for the management of feline sporotrichosis [4].

Intriguingly, KI has already been implicated in having an immunomodulatory effect [5,6,7,8] but the mechanisms through which this medication determines better outcomes in cats with sporotrichosis are yet to be confirmed.

However, despite the apparent susceptibility of cats to *Sporothrix* spp. infection and their important role as a source of infection, studies regarding immune response in feline sporotrichosis are scarce. The data already published show that, similarly to what is described for humans and mice, cell-mediated immunity seems to play a key role in the antifungal response in cats with sporotrichosis, given the association between CD4 levels and good outcomes and the granulomatous nature of the inflammatory response [1,9,10]. Still, phagocytes are stuffed with numerous yeast cells [9,10,11] with no efficient fungal clearance in most cases. This shows the importance of understanding the dynamics between *Sporothrix* yeast and the cat’s phagocytes.

The role of neutrophils in antifungal immunity has been raised in several fungal infections, including sporotrichosis, and comprises microbicidal mechanisms such as intracellular oxidative burst, degranulation, production of proinflammatory cytokines and neutrophil extracellular traps (NETs) [12,13,14,15,16,17]. This role of neutrophils can be demonstrated by increased susceptibility to fungal infections in individuals with neutropenia, knock-out models or primary immunodeficiencies involving neutrophil function, including chronic granulomatous disease [12,13,18]. Mice with chronic granulomatous disease develop systemic disease when infected with *Sporothrix* spp., which suggests that the metabolites produced by phagocytes play an important role in host protection against this fungus [13].

There are controversial findings regarding the role of neutrophils in sporotrichosis. In humans and mice, the number of neutrophils in tissue increases proportionally with the fungal burden [19,20,21,22] and it is directly related to patients with more lesions [21]. In feline sporotrichosis, an inverse relation is described between the intensity of neutrophil infiltrate and fungal burden [9,10]. Additionally, cats with sporotrichosis in good general condition frequently have skin lesions with marked infiltration of neutrophils [9]. However, the performance of neutrophils in lesions of feline sporotrichosis as well as its correlation with clinical outcome have been poorly investigated.

In this sense, the aim of this study is to evaluate for the first time the neutrophil oxidative burst in the peripheral blood of cats with sporotrichosis, using a flow cytometry-based assay. We also verify whether different profiles of oxidative burst can determine different treatment outcomes. Lastly, to investigate a potential effect of the treatment with KI on the neutrophil performance, we compare the neutrophil oxidative burst response in two treatment regimens: itraconazole alone and combination therapy of ITZ and KI.

## 2. Materials and Methods

### 2.1. Study Design and Procedures

Cats presented at the Laboratory of Clinical Research on Dermatozoonoses in Domestic Animals (Lapclin-Dermzoo), Evandro Chagas National Institute of Infectious Diseases (INI), Oswaldo Cruz Foundation (Fiocruz), Rio de Janeiro, Brazil, between September 2015 and July 2017, with clinical suspicion of sporotrichosis, were considered eligible.

The inclusion criteria for enrolment in the study were: diagnosis of sporotrichosis based on isolation and identification of *Sporothrix* spp. in culture from clinical samples and age between 1 and 8 years old. The exclusion criteria included previous antifungal or corticosteroid therapy.

Cats enrolled in the study were submitted to physical examination and divided into three groups, according to the distribution of skin lesions as previously established [23]. Group L1 comprised cats with skin lesions in one location; group L2 comprised cats with skin lesions in two non-contiguous locations and group L3 cats with skin lesions in three or more non-contiguous locations. These cats were randomly assigned to one of two possible treatment protocols: monotherapy with ITZ or combination therapy with ITZ and KI as described further in “Treatment Regimens”. The clinical condition was classified as good (absence of extracutaneous signs), fair (presence of clinical signs, such as mild inspiratory dyspnea, dehydration, pale mucosa and prostration) or poor (worsening of the clinical signs described in the fair group) [24].

Clinical follow-up appointments during the treatment were carried out every 4–6 weeks until an outcome event was established (clinical cure or treatment failure). Clinical cure was established when remission of all clinical signs was observed. Treatment failure was defined in two situations: 1—worsening of clinical signs and/or increase in the lesions in size and/or number at any time point during the follow-up (disease progression); 2—no improvement in healing of the lesions for two consecutive appointments (disease stagnation). Cats experiencing treatment failure were subsequently monitored as part of the routine follow-up at the Lapclin-Dermzoo.

Peripheral blood samples from enrolled cats were obtained at three time points during follow-up in order to assess neutrophil oxidative burst: T1 (before the beginning of the treatment); T2 (4–6 weeks of treatment); and T3 (outcome—clinical cure or treatment failure). The duration between T2 and T3 varied, depending on the time it took for an outcome event to occur.

All procedures performed on the cats, as well as the use of their biological samples, were approved by the Animal Use Ethics Committee, Fiocruz, under the license numbers LW-37/12 and LW-14/16. Prior to sample collection, the owners of the cats participating in this study provided signed informed consent forms.

### 2.2. Sample Collection

At the first time point (T1), sterile swabs were used to collect samples from exudates of skin ulcers to initiate the diagnosis of sporotrichosis through the isolation and identification of *Sporothrix* spp. in culture medium.

Peripheral blood samples were collected at time points T1, T2 and T3. Cats were sedated using intramuscular administration of 1% acepromazine maleate at 0.1 mg/kg and 10% ketamine hydrochloride at 10 mg/kg. Subsequently, approximately 1 mL of blood was collected in heparin tubes by jugular venepuncture for the assessment of neutrophil oxidative burst.

### 2.3. Treatment Regimens

As previously mentioned, cats enrolled in the study were randomly submitted to one of the following two therapeutic regimens:

1. Combination therapy: Oral administration of KI capsules (compounded medication), a dose of 2.5–10.0 mg/Kg/day, and ITZ capsules 100 mg (Prati-Donaduzzi^®^, Toledo, PR, Brazil), a dose of 100 mg/day [3].
2. Monotherapy: Oral administration of ITZ capsules 100 mg (Prati-Donaduzzi^®^, Toledo, PA, Brazil), 100 mg/day.

### 2.4. Neutrophil Oxidative Burst Test

A method using dihydrorhodamine 123 (DHR) oxidation and flow cytometry was employed to determine the presence of reactive oxygen intermediates during the oxidative burst in heparinized blood samples. The optimization of the test was based on the protocol previously described for feline peripheral blood [25].

To elicit neutrophil oxidative burst in peripheral blood samples, a non-specific stimulus involving Zymosan A particles (Sigma, St. Louis, MO, USA) and a specific stimulus using heat-killed yeasts from *Sporothrix* spp. were used. The neutrophil oxidative burst test was performed in the Laboratory of Immunoparasitology/Oswaldo Cruz Institute (IOC)/Fiocruz.

#### 2.4.1. Preparation of Heat-Killed Yeasts of *Sporothrix* spp.

*Sporothrix* spp. was isolated from a skin lesion of a cat presented at the Lapclin-Dermzoo. Cultures of *Sporothrix* spp. yeast cells were maintained in brain–heart infusion (BHI) broth at 35 °C with continuous agitation for a duration of five days. The inactivation of the fungus was performed as previously described [26]. Briefly, the yeast cells in BHI broth were heat-killed by incubation at 60 °C for one hour. Afterwards, the solution was submitted to three sequential washes with sterilized PBS and the pellet was resuspended in PBS. The concentration was adjusted to 1.0 × 10^10^/mL and aliquots of 100 μL were prepared for further opsonization. After the heat-killing procedure, one aliquot of heat-killed yeast was seeded on Sabouraud dextrose agar to assess viability and confirm the absence of fungal growth, indicating the successful heat inactivation of *Sporothrix* spp. yeasts.

#### 2.4.2. Opsonization Protocol

*Sporothrix* spp. (Sp) and Zymosan (Zym) were opsonized with cat normal serum (Abcam, Cambridge, MA, USA) before initiating the oxidative burst test. As previously described [25], Zym particles (20 mg) were opsonized in a solution of 2 mL of PBS and 1 mL of cat normal serum, through incubation for 1 h at 37 °C, under gentle agitation. Afterwards, particles were washed twice in PBS, resuspended in 4 mL of PBS at a concentration of 5 mg/mL and stored in aliquots at −20 °C. Shortly before use, the aliquots were thawed and diluted 1:1 in PBS for a final concentration of 2.5 mg/mL. Likewise, 10^9^ heat-killed yeasts of Sp (aliquots prepared as described above) were opsonized in a solution of 2 mL of PBS and 1 mL of cat normal serum, following the same protocol described for Zym. Aliquots of 50 μL containing ~10^7^ yeasts were prepared for use in the oxidative burst assay.

#### 2.4.3. Oxidative Burst Test Protocol

DHR (Sigma, St. Louis, MO, USA) was dissolved in DMSO (Sigma, St. Louis, MO, USA) at a concentration of 1 mM and stored in aliquots of 20 μL, at −20 °C. Immediately before use, an aliquot was thawed and diluted 1:10 in PBS for use in the stimulation protocol.

As the next step, 50 μL of heparinized blood from the cats was incubated with 100 μL of DHR for 15 min in a water bath at 37 °C, allowing for the incorporation of DHR by cells.

After that, the samples were stimulated with opsonized Sp (10^7^ heat-killed yeasts) or Zym (0.96 mg/mL) solutions by incubation in a water bath at 37 °C for 15 min. For each specimen, additional blood samples were incubated with DHR and without stimulation to assess neutrophil background activation under identical conditions. As a negative control, samples with no DHR and no stimulation were also included.

After stimulation, cells were fixed by incubating them with 66 μL of 7% formaldehyde in the dark at room temperature for 15 min, and red cells were subsequently lysed by the addition of 3 mL of distilled water. Cells were centrifuged at 200 g and resuspended in 400 μL of PBS for flow cytometry analysis.

#### 2.4.4. Flow Cytometry Analysis

All sample acquisitions were performed using the FACSCalibur flow cytometer (Becton Dickinson (BD, Franklin Lakes, NJ, USA)) from the Laboratory of Diagnostic Technology, Immunobiological Technology Institute, Fiocruz. The FACSCalibur was equipped with an argon laser (488 nm) and a diode red laser (635 nm) and DHR fluorescence was acquired through a 530/30 BP filter of the 488 nm laser. Both acquisition and analysis protocols were based on forward scatter (FSC) and side scatter (SSC) dot plots and on histograms generated by DHR fluorescence. A minimum of 10,000 events in the gate corresponding to granulocytes on the FSC × SSC dot plots were analyzed. Data acquisition and analyses were performed using CellQuest software version 5.2.1 (BD, Franklin Lakes, NJ, USA) and FlowJo software version 7.6.5 (BD, Franklin Lakes, NJ, USA), respectively.

The results were presented as the percentage of positive cells (Supplementary Figure S1) or by stimulation index (SI) for each stimulant. The percentage of positive cells was obtained by subtracting the percentage of background activation from the percentage of positive granulocytes in the stimulated specimen. The SI was defined as the ratio of the geometric mean fluorescence intensity of stimulated samples (using either Zymosan or *Sporothrix* spp.) to that of background activation/non-stimulated samples (BA).

### 2.5. Data Analysis

Data were compiled and analyzed by the Statistical Package for Social Sciences (SPSS, INC, Chicago, IL, USA), version 16.0. The Pearson’s chi-square test for independence (pP) was used to determine a significant association between categorical variables, while Fisher’s exact test (pFET) was applied for comparing frequencies in variables with only two categories. The Kruskal–Wallis (pKW) and Mann–Whitney (pMW) tests were used to test for statistical differences in neutrophil oxidative burst parameters and categorical variables at each time point. The Friedman test (pF) was used to determine statistically significant changes over time. A Pearson correlation (pPC) was performed to assess the relationship between age and oxidative burst levels. Odds ratios (ORs) and their 95% confidence intervals (CIs) were obtained to evaluate the impact of different variables on clinical cure. *p*-values < 0.05 were considered significant in all analyses.

## 3. Results

### 3.1. Sample Characteristics

**Figure 1:**
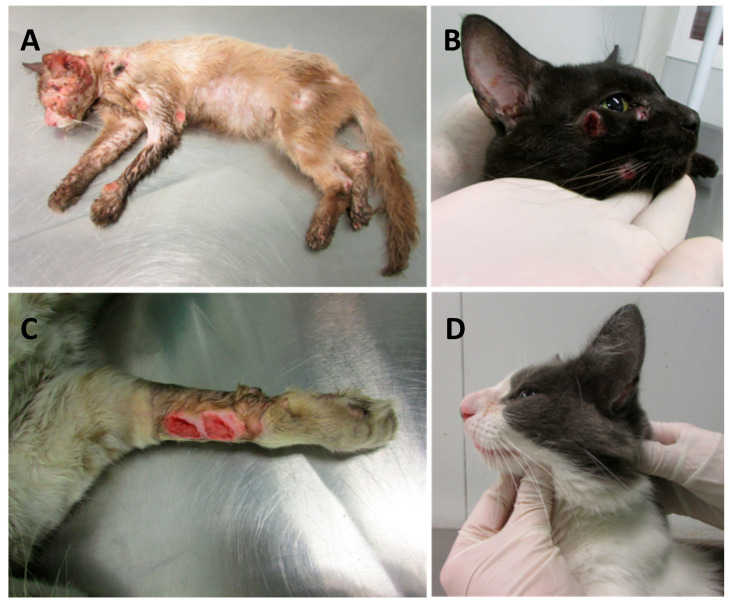
Feline sporotrichosis. Skin lesions in cats presented at Lapclin-Dermzoo/INI/Fiocruz, Rio de Janeiro, Brazil. Disseminated skin lesions (**A**); Multiple skin ulcers on the face (**B**); Skin ulcers on the forelimb (**C**); Swelling over the bridge of the nose (**D**).

Forty-seven cats with sporotrichosis were enrolled in the study (Supplementary Table S1). Molecular identification was performed in *Sporothrix* isolates from 32 cats through PCR T3B fingerprinting [27] and all of them were identified as *S. brasiliensis*. The age of the cats ranged from 12 to 96 months (median = 24 months). All cats showed skin lesions and 20 (42.6%) also showed lesions in nasal mucosa (Figure 1A–D). Most of the cats were categorized in group L3 (63.8%; Supplementary Table S1). All cats included in groups L1 and L2 were in good general condition (pP = 0.017). In neutered/spayed cats, good general condition was significantly more frequent (pFET = 0.004) and respiratory signs were significantly less frequent (pFET = 0.049) compared to non-neutered cats.

### 3.2. Treatment Follow-Up

**Figure 2:**
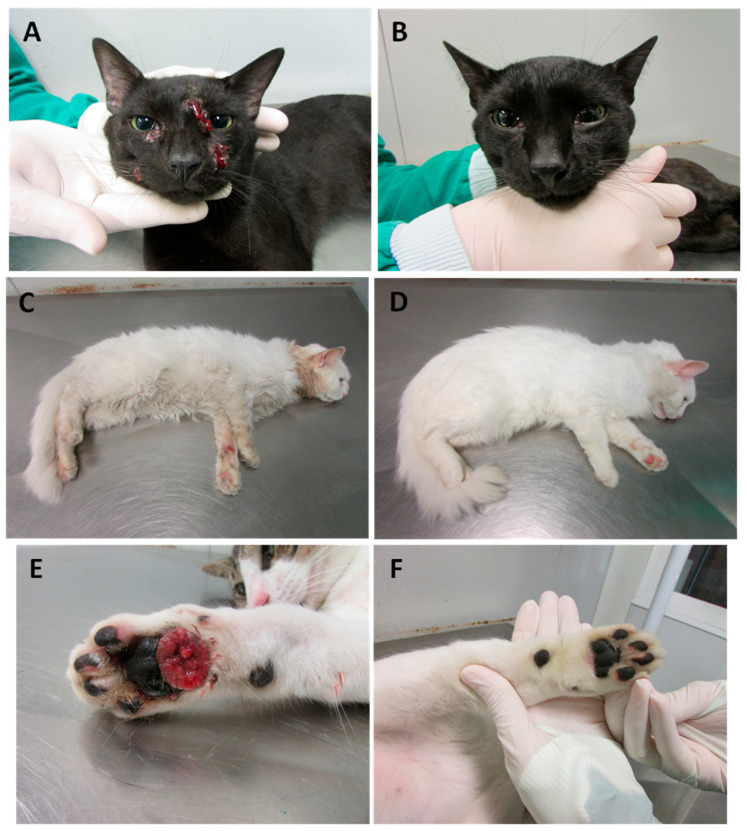
Feline sporotrichosis. Response to treatment. Clinical cure. Cat presenting multiple skin ulcers on the face before the beginning of the treatment (**A**) and complete healing after eighteen weeks of treatment with the combination of itraconazole and KI (**B**); Cat presenting swelling over the bridge of the nose and a skin ulcer at the palmar side of the left forelimb before the beginning of the treatment (**C**) and complete healing and the remission of clinical signs after twenty-nine weeks of treatment with itraconazole (**D**); Cat presenting ulcerated lesion on the right forelimb pad before the beginning of the treatment (**E**) and complete healing after sixteen weeks of treatment with itraconazole (**F**).

**Figure 3:**
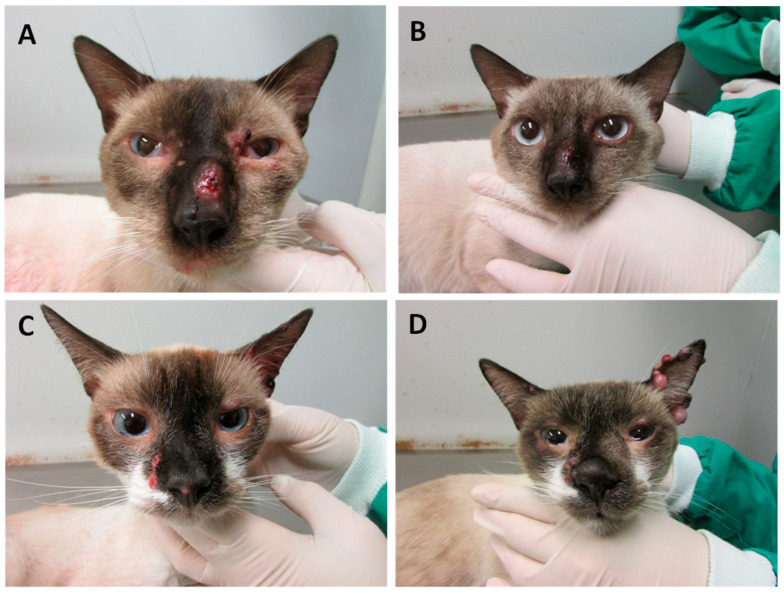
Feline sporotrichosis. Response to treatment. Treatment failure. Cat presenting swelling and ulceration on the bridge of the nose and periocular skin lesions before the beginning of the treatment (**A**); and the persistence of the skin lesion on the bridge of the nose after eight weeks of treatment with itraconazole (**B**); Cat presenting skin ulcer lateral to the nose and nasal planum before the beginning of the treatment (**C**); and persistence of the lesion with the swelling of the nose and the appearance of multiple nodules on the left ear after sixteen weeks of treatment with the combination of itraconazole and KI (**D**).

Among the 47 cats included in the treatment study, 36 were followed up until a clinical outcome—14 of them were treated with ITZ monotherapy and 22 with a combination of ITZ and KI capsules. Eleven cats have not returned for the follow-up appointments. Clinical cure (Figure 2A–F) occurred in twenty-seven (75.0%) cats and therapeutic failure (Figure 3A–D) in nine (25.0%; Supplementary Table S2).

Good general condition before the beginning of the therapy (T1) was significantly more frequent (pFET = 0.05; OR 6.4, 95% CI 1.3–29.9) in cats that were cured (24/27) in comparison to cats that had therapy failure (5/9). Clinical cure was also associated with the absence of nasal mucosa involvement: 3/17 cats with clinical cure had involvement of the nasal mucosa versus 7/9 cats with treatment failure (pFET = 0.005; OR 12.3, 95% CI 2.1–4.3). All cats within the group L1 (*n* = 5) presented clinical cure.

With regard to treatment regimens, the cure rate was 50.0% (7/14) in cats under treatment with ITZ monotherapy and 90.9% (20/22) in cats treated with the combination of drugs (pFET = 0.014). Treatment length until clinical cure ranged from 13 to 31.4 weeks (median = 17.7 weeks) in cats treated with ITZ monotherapy and from 8 to 44.6 (median = 16.9 weeks) in cats treated with the combination therapy. There was no significant difference in the timespan to cure between the two treatments in cats that showed cure.

Amongst cats that had treatment failure (*n* = 9), there was a clear distinction in the course of disease during the treatment: in most of these animals (6/9), treatment failure was determined due to disease stagnation; three animals, two treated with ITZ+KI and one with ITZ monotherapy, developed severe disease progression.

Nasal mucosa lesions and/or respiratory signs were associated with disease stagnation when compared to cured cats (pFET = 0.001 and 0.01, respectively) and to cats with disease progression (pP = 0.048 and 0.083, respectively). All (*n* = 6) cats with disease stagnation presented nasal mucosa lesions and 5/6 presented respiratory signs.

Two of the cats with severe disease died of sporotrichosis and one was submitted to euthanasia on welfare grounds.

### 3.3. Neutrophil Oxidative Burst Test

This study was split into two stages: cross-sectional and longitudinal. In the cross-sectional stage, samples from cats with sporotrichosis before the beginning of the antifungal therapy were evaluated. The longitudinal stage compared data across the treatment follow-up and against the clinical outcome with each treatment regimen.

#### 3.3.1. Cross-Sectional Analysis (*n* = 47)

The neutrophil oxidative burst test was performed at T1 in all cats included in this study (*n* = 47) and the results were compared against the demographic and clinical variables (Supplementary Table S3). In four cats, the analysis was not performed in at least one of the parameters due to insufficient cell recovery.

**Figure 4:**
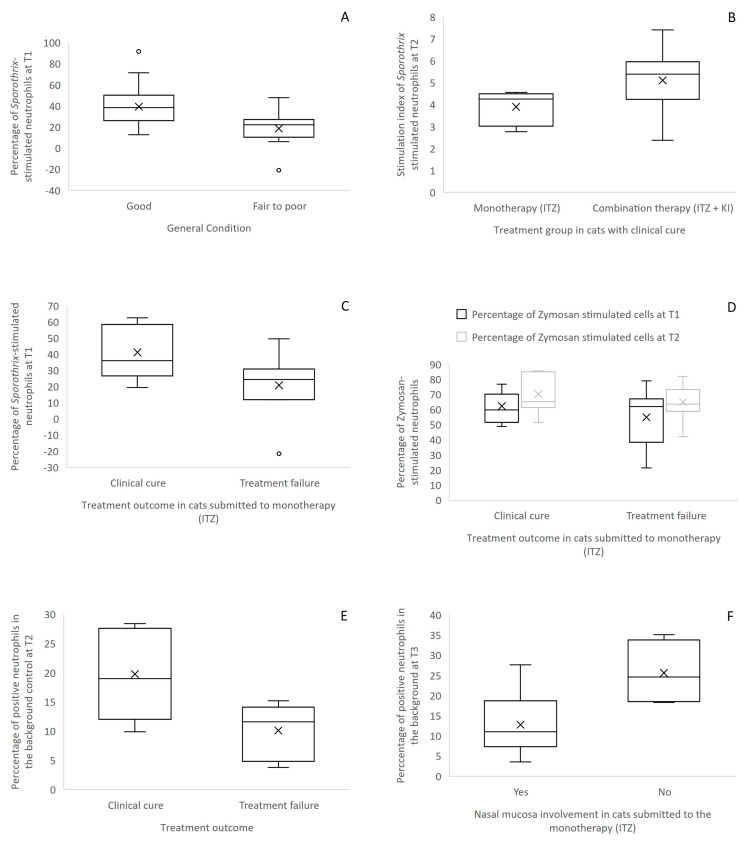
The neutrophil oxidative burst test performed on the peripheral blood of cats with sporotrichosis demonstrates a significant variation in some neutrophil oxidative burst parameters, depending on clinical and therapeutic features. Good general condition is associated (pMW = 0.003) with a higher percentage of *Sporothrix*-stimulated neutrophils at T1 (**A**); Clinical cure with combination therapy is associated (pMW = 0.035) with a higher stimulation index in *Sporothrix*-stimulated neutrophils at T2 in comparison to monotherapy (**B**); Clinical cure with monotherapy is associated (pMW = 0.05) with a higher percentage of *Sporothrix*-stimulated neutrophils at T1 in comparison to treatment failure (**C**); The percentage of Zymosan-stimulated neutrophils is significantly higher (pF = 0.014) in T2 in comparison to T1 in clinical cure in comparison to treatment failure (**D**); The percentage of positive neutrophils in the background control at T2 is significantly higher (pMW = 0.03) in clinical cure with monotherapy in comparison to treatment failure (**E**); Nasal mucosa involvement is associated (pMW = 0.02) with a lower percentage of positive neutrophils in the background control at T3 in cats submitted to the monotherapy (**F**).

Regarding the percentage of stimulated neutrophils, cats in good general condition showed a significantly (pMW = 0.003) higher percentage of Sp-stimulated neutrophils at T1 in comparison to those in fair to poor condition (Figure 4A). When it comes to neutrophil oxidative burst SI, there was no association with any variable in the cross-sectional analysis.

#### 3.3.2. Longitudinal Analysis/Treatment Follow-Up (*n* = 32)

From the 36 cats that were followed up until a treatment outcome, the longitudinal analysis of the neutrophil oxidative burst was performed in 32 (Supplementary Table S4).

In animals that had clinical cure as the treatment outcome, a significantly higher SI in Sp-stimulated neutrophils (pMW = 0.035) was detected at T2 in the group receiving the combination therapy (Figure 4B). We also observed a positive correlation between clinical cure and Sp-stimulated neutrophils’ response in animals submitted to the treatment with ITZ alone, with a higher percentage of Sp-stimulated neutrophils at T1 being associated (pMW = 0.05) with clinical cure in comparison to treatment failure (Figure 4C). Also, in the group treated with ITZ alone, a significant increase in the percentage of Zym-stimulated neutrophils from T1 to T2 was detected in cured animals (pF = 0.014) in comparison to treatment failure (Figure 4D).

The percentage of positive cells in the background controls ranged from 0.11 to 63.2 (median = 11.7) at T1, 2.63 to 38.3 (median = 14.7) at T2 and 3.7 to 35.1 (median = 13) at T3. At T2, the percentage of positive neutrophils in the background control was significantly higher (pMW = 0.03) in cured animals in comparison to animals in treatment failure related to the monotherapy (Figure 4E). In animals submitted to the monotherapy, the percentage of positive neutrophils in the background control at T3 was significantly lower (pMW = 0.02) in animals with nasal mucosa involvement in comparison to those that did not present nasal mucosa involvement (Figure 4F).

## 4. Discussion

The present study for the first time sheds some light on the association between neutrophil oxidative burst and the outcome of sporotrichosis in cats. Our results suggest a beneficial role of neutrophils in feline sporotrichosis, with a higher percentage of *Sporothrix*-stimulated neutrophils in cats in good general condition. Also, higher percentages of activated neutrophils before (T1) and early in the treatment (T2) were related to clinical cure after stimulation with *Sporothrix* spp. and Zymosan, respectively. Lower percentages of neutrophils in the background control at T3 were related to cats with nasal mucosa involvement, which is usually associated with clinical failure. These findings demonstrate a positive correlation between neutrophil activation and the cure process in feline sporotrichosis.

Although the role of neutrophils in the anti-*Sporothrix* immune response has been little explored, lesions of feline sporotrichosis with more neutrophils were shown to be related to better outcomes and reduced fungal load as opposed to lesions with scarce neutrophilic response, in which a high fungal load is usually found [9,10]. Interestingly, in humans, the fixed form of sporotrichosis, which is more benign, produces lesions with fewer neutrophils in comparison to the lymphocutaneous form, considered more severe [21]. The neutrophil-associated tissue destruction is believed to facilitate lymphatic dissemination of fungal cells in the lymphocutaneous form of sporotrichosis [28]. Likewise, histologically, neutrophils and liquefaction are more likely to be present in lesions of human sporotrichosis in which the fungal agent is visualized, implying a higher fungal burden in those lesions [22]. The controversial observations in human and feline sporotrichosis are not entirely unexpected, given the typical disparity in inflammatory response and fungal burdens in their respective lesions.

Activation of pattern recognition receptors (PRRs) by pathogen-associated molecular patterns (PAMPs) determines signal transduction pathways that tailor the type of inflammatory response and can ultimately dictate neutrophil microbicidal mechanisms [16,18]. When it comes to *Sporothrix* spp. infection, neutrophils could be activated differently in humans and cats, leading to distinct microbicidal mechanisms and inflammation profiles. Although many PRRs have been described as participating in anti-*Sporothrix* response in humans and mice, we do not know which receptors and associated pathways are involved in the recognition of *Sporothrix* PAMPs by feline neutrophils or peripheral blood mononuclear cells (PBMCs). Toll-like receptors (TLRs) were described to be involved in the response to *Aspergillus* and *Microsporum* in cats [9,29,30]. However, it is noteworthy that, unlike *Sporothrix*, these fungal species typically generate hyphae in the host. The larger size of hyphae, in comparison to yeast, appears to trigger different mechanisms of killing, usually involving NETs [31,32]. NETs have been described in response to *Sporothrix* [21,33,34], but it is not clear whether they play a significant role in *Sporothrix* clearance. Interestingly, NET formation was reported to occur more frequently in the lymphocutaneous form of human sporotrichosis compared to the fixed cutaneous form [21]. However, further studies are necessary to better understand their role in each type of lesion. Diverse fungal morphologies, such as yeast, conidia or hyphae, can express different virulence factors and be recognized by distinct receptors, thereby eliciting different responses, ranging from the recruitment of neutrophils to the induction of a Th2-driven response [35]. Recognition of *Sporothrix* PAMPs by the complement protein C3 with subsequent complement activation has been described as an important step to promote *Sporothrix* opsonization and phagocytosis by human macrophages [36] and could be further explored as a mechanism in cats as well.

The flow cytometric method utilized in our study effectively detected the neutrophil oxidative burst in the peripheral blood of cats. Zymosan was used as a positive control since it is considered a potent neutrophil stimulator and as such was expected to produce more stimulation than *Sporothrix* spp. During an infection such as with *Sporothrix* spp., a proinflammatory state promotes pre-activation—or priming—of circulating neutrophils [37], which is needed for their full activation and production of reactive oxygen species (ROS) in the oxidative burst. Neutrophils previously primed in circulation exhibit a stronger and quicker oxidative burst upon encountering a second stimulus [38], both in vivo and in vitro, as opposed to “resting” neutrophils. This heightened responsiveness could lead to their potential exhaustion [37]. In feline sporotrichosis, before the start of the treatment, neutrophilia is more common [23] and the fungal load is usually higher [10,39]. In this scenario, a higher number of neutrophils would be expected to be already primed in the blood by chemotactic factors and fungal antigens. Consequently, they may be more prone to exhaustion upon additional stimulation. We hypothesize that in our study a large number of primed neutrophils at T1 could have undergone exhaustion due to the strong in vitro stimulation with Zymosan. As a result, some of these neutrophils could have perished during the processing, eluding detection in the assay. This would explain the fact that high levels of Zymosan-stimulated neutrophils in this study were associated with clinical cure at T2 but not at T1.

Excessive sample manipulation can also induce in vitro activation of neutrophils even in the absence of priming or in vitro stimulants [40]. In our study, we minimized sample manipulation by utilizing whole blood specimens and omitting centrifugation steps. Thus, we presume that neutrophils present in the background controls were already primed in the circulation, making them more susceptible to activation even with minimal manipulation.

Altogether, we hypothesize that chemotactic factors in the blood could have promoted early neutrophil priming and been beneficial for the host. On the other hand, a lack of stimulation in some cats could have prevented neutrophils from being efficiently recruited and activated. The lack of activation could be a result of defective pathways in the host’s innate response or of fungal mechanisms of evasion, culminating in inappropriate signaling. For example, increased fungal burden and reduction in neutrophil recruitment in TLR4−/− mice have been attributed to the decrease in cytokine production due to the lack of TLR activation [41], which reinforces the importance of the early steps of host–pathogen interaction in the overall response to *Sporothrix* spp. Another possibility is a scarce neutrophilic response related to immune exhaustion in some cats, preventing the appropriate signaling from being triggered. Chronic or excessive antigen exposure has been shown to upregulate immune exhaustion pathways and be detrimental to patients and mouse models with invasive fungal infections such as histoplasmosis [42,43]. Conversely, the blockade of these pathways seems to cause significant clinical improvement, reduce the fungal burden and increase the number of intralesional neutrophils [42,44]. The correlation between immune exhaustion and severe sporotrichosis has been previously raised by this group [24] particularly when there is co-infection by feline immunodeficiency virus (FIV), but further investigation is pending.

*Sporothrix*-related factors, such as the sugar composition of the fungal wall, production of melanin and antioxidant enzymatic system, were shown to protect the fungal cell against host phagocytes early in their interaction [45,46,47,48,49,50,51,52]. The interaction between *S. brasiliensis*, enrolled as a highly pathogenic species [53,54,55], and human PBMCs leads to increased levels of IL-10 in comparison to *S. schenckii* sensu stricto, and there seems to also be a variable cytokine response depending on which PRR is being activated [46]. High IL-10 levels could potentially induce a decreased phagocytic activity in neutrophils, leading to an inadequate host response [37]. On the other hand, another study found a more pronounced proinflammatory profile induced by *S. brasiliensis* in human PBMCs in comparison to *S. schenckii* [56], suggesting that factors within the same species can impact their behavior upon interaction with the host. The study by Corrêa-Moreira et al. [57] indicates that virulence may be related to the isolate independent of the *Sporothrix* species. *Sporothrix* virulence characteristics have not been assessed in the causative isolates from this study, but it is safe to say that evasion mechanisms of the fungus may interfere with the capacity of the feline host to cope with the infection, and it is possible that the impaired phagocytosis in cats with sporotrichosis may be related to more pathogenic isolates of *Sporothrix*.

Our results also endorse the use of the combination therapy with ITZ and KI as an excellent alternative for the treatment of feline sporotrichosis, as previously presented [3,4,58,59,60]. Although the combination therapy led to significantly increased cure rates of feline sporotrichosis in this study, it was not enough to successfully treat cats with progressive disease. On the other hand, since failure by disease stagnation did not occur in the group treated with the combination therapy, this therapeutic protocol seems appropriate for cases that stagnate using ITZ alone, which seems to be the case when clinical cure is achieved after introducing KI to ITZ-refractory sporotrichosis in cats [58]. Our results suggest that most cats failing to respond to monotherapy are more likely to develop a chronic, persistent disease rather than experiencing a progressive and rapid fatal clinical course.

The correlation between oxidative burst and successful treatment with the use of KI could not be properly assessed in this study, considering there were only two failures in this treatment group, and they both seemed to be of a less common presentation, with dramatic progression into severe disease despite treatment. Thus, it is not possible to rule out an influence of KI on the oxidative burst and the outcome, especially considering that, in cured cats, after the beginning of the treatment (T2), KI-treated cats presented a higher SI in *Sporothrix*-stimulated neutrophils in comparison to cats submitted to monotherapy. No other difference between the two treatment groups was detected, suggesting that the increased levels of activated neutrophils in the monotherapy related to cure were comparable to the levels found in the combination therapy.

It is noteworthy that reactive oxygen intermediates produced during phagocytes’ oxidative burst may convert iodides in iodine, which has been reported to have a microbicidal role, including efficacy against fungal cells [61,62,63]. This may be a possible explanation for the mechanisms of fungal clearance and favorable outcomes in cats with sporotrichosis that are given oral KI, especially considering the apparent beneficial role of neutrophil oxidative burst and the excellent cure rates of the combination therapy. Interestingly, studies in humans show that excessive iodine intake may contribute to the onset of autoimmune thyroiditis, and this correlation has been linked to high levels of circulating proinflammatory IL-17 [64,65,66]. The occurrence of similar changes in cats receiving KI treatment remains uncertain. However, an increase in IL-17 could play a positive role in the anti-*Sporothrix* response, potentially elucidating the positive outcomes associated with this treatment. For more in-depth understanding, periodical monitoring of thyroid function and investigation of the Th17 response in treated cats would be necessary. Unexpectedly, early studies in humans indicated that employing iodides for the treatment of sporotrichosis does not appear to enhance the efficiency of phagocytosis [67]. Instead, it seems to reduce the chemotaxis of neutrophils [68] and the synthesis of reactive oxygen species by these cells [69]. These contradictory findings reflect the existing gap in our understanding of KI mechanisms and emphasize the need for further studies.

Although this study has brought new information on the yet unclear mechanisms driving the unsuccessful host response in lesions of feline sporotrichosis, the current findings could only scratch the surface of the complex host–pathogen interactions between the feline immune system and *Sporothrix*. Further investigation is necessary to understand host and pathogen roles in the outcome of this *Sporothrix*–cat interaction. The association of oxidative burst performance in neutrophils and different cytokine expression could help to determine the dominant response in each situation, but the factors involved in the induction of one type or another of immunity would still need to be explored. Therefore, we encourage further studies to put all these variables together and warrant a more accurate understanding of their interplay and to drive the development of future therapeutic interventions.

The oxidative burst test could also be further investigated as a tool for prognosis assessment in such a way that those cats in which an optimal activation of neutrophils is not detected before the treatment would be more likely to fail to cure with treatment with ITZ alone. Additional studies would need to be carried out to establish accurate threshold values for this test in order to understand the behavior of these parameters in cats. In this context, the determinant role of KI and its potential interplay with neutrophil recruitment and activation still need additional elucidation.

## Data Availability

Data are contained within the article.

## Supplementary Materials

The following supporting information can be downloaded at: https://www.mdpi.com/article/10.3390/jof10060422/s1, Figure S1: Representative analysis of neutrophil activation in peripheral blood of cats with sporotrichosis. Forward scatter (FSC-H) vs. Side Scatter (SSC-H) dot plot showing the cell gate (A). Histograms of fluorescence after incubation with 60 µL of the Zymosan solution (~0.96 mg/mL) (B) and 107 heat-killed yeasts of Sporothrix spp. for 15 min (C); without DHR and without stimulation/negative control (D) and with only DHR but without stimulation/background activation (E). The horizontal axis (X) represents the fluorescence intensity of DHR and the vertical axis (Y) the number of cells. The cursor shows the number of DHR-positive cells inside cell gate; Table S1: Demographic and clinical characteristics of 47 cats with sporotrichosis presented at Lapclin-Dermzoo/INI/Fiocruz, Rio de Janeiro, between September 2015 and July 2017; Table S2: Frequencies of clinical–epidemiological data from 36 cats with sporotrichosis, according to clinical outcome of different therapeutic regimens; Table S3: Descriptive analysis of oxidative burst parameters in cats with sporotrichosis before the beginning of the treatment (T1) according to demographic and clinical variables. Rio de Janeiro (September/2015–July/2017); Table S4: Descriptive analysis of oxidative burst parameters at the three different timepoints in cats with sporotrichosis according to treatment group and outcome. Rio de Janeiro (September/2015–July/2017).
