# Supplementary material for: Neutrophil Oxidative Burst Profile Is Related to a Satisfactory Response to Itraconazole and Clinical Cure in Feline Sporotrichosis
Source: J Fungi (Basel). 2024 Jun 14;10(6):422. doi: 10.3390/jof10060422 (PMC11205038; doi:10.3390/jof10060422)
Supplement: Supplementary file 1 [file jof-10-00422-s001.zip › Supplementary Figure S1.pdf]

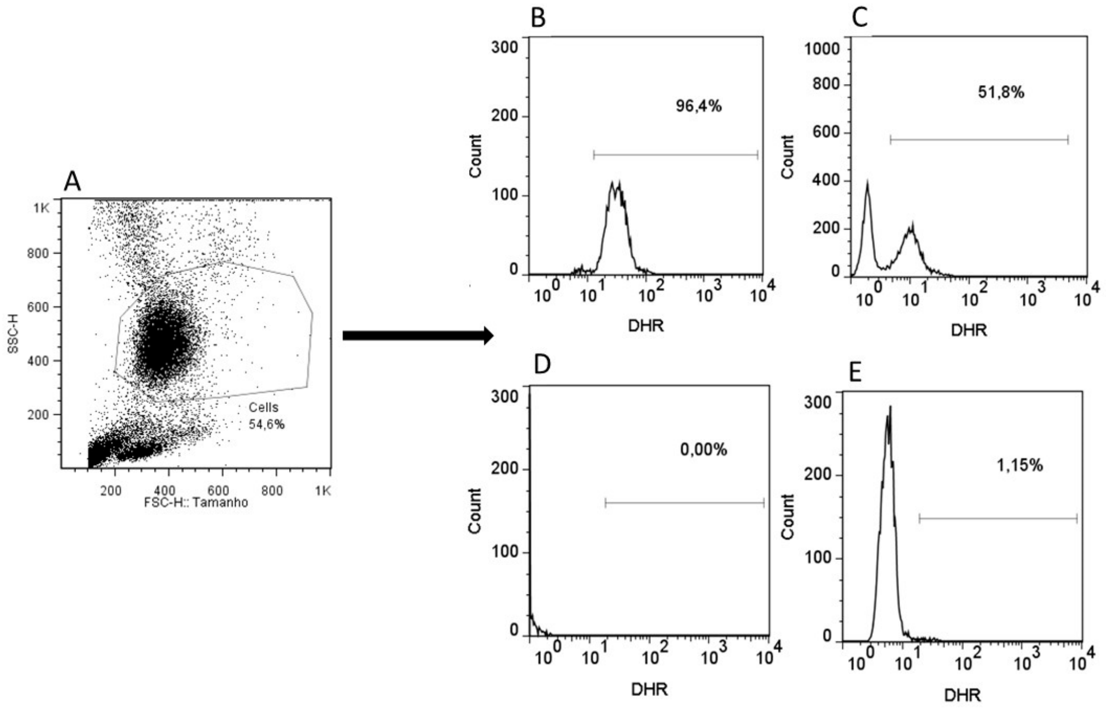

Supplementary Figure S1. Representative analysis of neutrophil activation in peripheral blood of cats with sporotrichosis. Forward scatter (FSC-H) vs. Side Scatter (SSC-H) dot plot showing the cells gate (A). Histograms of fluorescence after incubation with 60 µL of the Zymosan solution (~0.96 mg/mL) (B) and 10^7 heat-killed yeasts of *Sporothrix* spp. for 15 minutes (C); without DHR and without stimulation/negative control (D) and with only DHR, but without stimulation/background activation (E). The horizontal axis (X) represents the fluorescence intensity of DHR and the vertical axis (Y) the number of cells. The cursor shows the number of DHR-positive cells inside cells gate.
