# Supplementary material for: Neutrophil Oxidative Burst Profile Is Related to a Satisfactory Response to Itraconazole and Clinical Cure in Feline Sporotrichosis
Source: J Fungi (Basel). 2024 Jun 14;10(6):422. doi: 10.3390/jof10060422 (PMC11205038; doi:10.3390/jof10060422)
Supplement: Supplementary file 1 [file jof-10-00422-s001.zip › Supplementary Table S1.pdf]

Supplementary table S1: Demographic and clinical characteristics of 47 cats with sporotrichosis presented at Lapclin-Dermzoo/INI/Fiocruz, Rio de Janeiro, between September 2015 and July 2017.

Variable	Number of cats n (%)
**Sex**	
Male	41 (87.2)
Female	6 (12.8)
**Neutering/Spaying status**	
Yes	23 (48.9)
No	24 (51.1)
**General condition**	
Good	36 (76.6)
Fair to poor	11 (23.4)
**Distributions of lesions**	
L1	8 (17.0)
L2	9 (19.1)
L3	30 (63.8)
**Nasal mucosa involvement**	
Yes	20 (42.6)
No	27 (57.4)
**Respiratory signs**	
Yes	13 (27.7)
No	34 (72.3)

L1: Cats with lesions in one location; L2: Cats with lesions in two non-contiguous locations; L3: Cats with lesions in three or more non-contiguous locations
