# Supplementary material for: Neutrophil Oxidative Burst Profile Is Related to a Satisfactory Response to Itraconazole and Clinical Cure in Feline Sporotrichosis
Source: J Fungi (Basel). 2024 Jun 14;10(6):422. doi: 10.3390/jof10060422 (PMC11205038; doi:10.3390/jof10060422)
Supplement: Supplementary file 1 [file jof-10-00422-s001.zip › Supplementary Table S2.pdf]

Supplementary table S2. Frequencies of clinical-epidemiological data from 36 cats with sporotrichosis, according to clinical outcome of different therapeutic regimens.

Variable	Therapeutic regimen			
	ITZ		ITZ + KI	
	Clinical cure (N = 7) n (%)	Treatment failure (N = 7) n (%)	Clinical cure (N = 20) n (%)	Treatment failure (N = 2) n (%)
**Sex**				
Male	7 (50.0%)	6 (42.9%)	14 (77.8%)	2 (11.1%)
Female	0 (0.0%)	1 (7.1%)	2 (11.1%)	0 (0.0%)
**Neutering/Spaying**				
Yes	4 (28.6%)	3 (21.4%)	10 (45.5%)	1 (4.5%)
No	3 (21.4%)	4 (28.6%)	10 (45.5%)	1 (4.5%)
**General condition**				
Good	7 (50.0%)	4 (28.6%)	17 (77.3%)	1 (4.5%)
Fair to poor	0 (0.0%)	3 (21.4%)	3 (13.6%)	1 (4.5%)
**Distribution of lesions**				
L1	2 (14.3%)	0 (0.0%)	3 (13.6%)	0 (0.0%)
L2	3 (21.4%)	1 (7.1%)	3 (13.6%)	0 (0.0%)
L3	2 (14.3%)	6 (42.9%)	14 (63.6%)	2 (9.1%)
**Nasal mucosa involvement**				
Yes	3 (21.4%)	7 (50.0%)	3 (13.6%)	0 (0.0%)
No	4 (28.6%)	0 (0.0%)	17 (77.3%)	2 (9.1%)
**Respiratory signs**				
Yes	3 (21.4%)	5 (35.7%)	3 (13.6%)	0 (0.0%)
No	4 (28.6%)	2 (14.3%)	17 (77.3%)	2 (9.1%)

L1: Cats with lesions in one location; L2: Cats with lesions in two non-contiguous locations; L3: Cats with lesions in three or more non- contiguous locations.
ITZ=Itraconazole; KI=Potassium iodide.
