# Supplementary material for: Neutrophil Oxidative Burst Profile Is Related to a Satisfactory Response to Itraconazole and Clinical Cure in Feline Sporotrichosis
Source: J Fungi (Basel). 2024 Jun 14;10(6):422. doi: 10.3390/jof10060422 (PMC11205038; doi:10.3390/jof10060422)
Supplement: Supplementary file 1 [file jof-10-00422-s001.zip › Supplementary Table S3.pdf]

Supplementary Table S3: Descriptive analysis of oxidative burst parameters in cats with sporotrichosis before the beginning of the treatment (T1) according to demographic and clinical variables. Rio de Janeiro (September/2015-July/2017).

Variable		Oxidative burst parameters				
		**SI-Zym**	**SI-Sp**	**%RHD**	**%Zym**	**%Sp**
**Sex**						
Male	Median	9.2	5.9	11.8	63.7	30.5
	Minimum	3.0	2.5	0.41	21.4	-21.0
	Maximum	23.0	9.6	63.2	92.0	91.6
	N	41	37	41	40	38
Female	Median	11.4	6.0	7.8	71.7	45.2
	Minimum	3.6	3.0	0.11	67.0	23.6
	Maximum	24.5	10.1	19.4	91.8	62.3
	N	6	6	6	6	6
**Neutered/Spayed**						
Yes	Median	9.2	5.9	11.7	68.5	36.0
	Minimum	3.0	2.5	0.1	36.0	6.2
	Maximum	24.5	10.1	63.2	91.8	91.6
	N	23	21	23	22	21
No	Median	9.0	5.9	10.9	61.2	30.2
	Minimum	3.3	2.7	0.4	21.4	-21.0
	Maximum	23.0	9.6	58.5	92.0	71.5
	N	24	22	24	24	23
**General condition**						
Good	Median	9.8	6.5	11.8	68.1	38.4
	Minimum	3.0	2.5	0.1	36.0	12.7
	Maximum	24.5	10.1	63.2	91.8	91.6
	N	36	33	36	35	34
Fair to poor	Median	7.1	4.3	10.2	56.0	22.2
	Minimum	3.3	2.7	4.0	21.4	-21.0
	Maximum	18.3	8.2	58.5	92.0	47.7
	N	11	10	11	11	10

**Distribution of lesions**						
L1	Median	11.2	5.9	8.9	68.1	24.5
	Minimum	3.0	2.5	3.0	48.8	13.5
	Maximum	16.4	7.3	15.2	76.3	58.4
	N	8	6	8	7	7
L2	Median	9.2	4.8	17.2	69.0	36.0
	Minimum	3.1	2.6	2.5	51.4	23.6
	Maximum	15.4	8.5	35.2	88.8	54.0
	N	9	9	9	9	9
L3	Median	8.3	6.4	11.6	63.2	29.8
	Minimum	3.3	2.7	0.1	21.4	-21.0
	Maximum	24.5	10.1	63.2	92.0	91.6
	N	30	28	30	30	28
**Nasal mucosa involvement**						
Yes	Median	9.5	6.5	12.3	63.2	30.2
	Minimum	3.1	2.6	0.1	21.4	-21.0
	Maximum	24.5	10.1	58.5	92.0	62.5
	N	20	19	20	20	19
No	Median	9.2	5.5	11.5	68.9	34.1
	Minimum	3.0	2.5	2.5	34.6	6.2
	Maximum	19.4	9.6	63.2	90.4	91.6
	N	27	24	27	26	25
**Respiratory signs**						
Yes	Median	8.5	5.5	12.6	60.8	28.6
	Minimum	3.1	2.6	0.1	21.4	-21.0
	Maximum	24.5	10.1	58.5	92.0	62.5
	N	13	12	13	13	12
No	Median	9.4	5.9	10.9	68.1	33.7

	Minimum	3.0	2.5	0.4	34.6	6.2
	Maximum	19.4	9.6	63.2	90.4	91.6
	N	34	31	34	33	32

L1: lesions in one location; L2: lesions in two non-contiguous locations; L3: lesions in three or more non-contiguous locations

SI-Zym: Stimulation index of Zymosan stimulated cells; SI-Sp: Stimulation index of *Sporothrix* stimulated cells; %RHD: percentage of basal activation; %Zym: Percentage of Zymosan stimulated cells; %Sp: Percentage of *Sporothrix* stimulated cells
