# Supplementary material for: Neutrophil Oxidative Burst Profile Is Related to a Satisfactory Response to Itraconazole and Clinical Cure in Feline Sporotrichosis
Source: J Fungi (Basel). 2024 Jun 14;10(6):422. doi: 10.3390/jof10060422 (PMC11205038; doi:10.3390/jof10060422)
Supplement: Supplementary file 1 [file jof-10-00422-s001.zip › Supplementary Table S4.pdf]

Supplementary Table S4: Descriptive analysis of oxidative burst parameters at the three different timepoints in cats with sporotrichosis according to treatment group and outcome. Rio de Janeiro (September/2015-July/2017).

			Oxidative burst parameters at each timepoint														
			**T1**					**T2**					**T3**				
**Treatment group**	**Outcome**		**SI-Zym**	**SI-Sp**	**%RHD**	**%Zym**	**%Sp**	**SI-Zym**	**SI-Sp**	**%RHD**	**%Zym**	**%Sp**	**SI-Zym**	**SI-Sp**	**%RHD**	**%Zym**	**%Sp**
**Monotherapy (ITZ)**	**cure**	Median	6.7	4.3	13.0	59.7	36.0	6.4	4.3	19.0	65.3	35.6	7.1	4.4	19.0	69.8	45.5
		Minimum	3.0	2.5	3.8	48.8	19.4	3.8	2.8	9.9	51.4	22.2	4.4	3.8	3.6	43.6	22.5
		Maximum	14.8	7.8	35.2	77.0	62.5	7.8	4.6	28.4	85.6	55.5	12.9	5.8	35.1	79.5	65.1
		N	7	7	7	7	7	7	7	7	7	7	7	7	7	7	7
	**failure**	Median	8.5	6.5	11.9	61.9	24.4	8.2	4.7	11.6	63.6	45.3	6.9	4.8	11.4	73.9	45.4
		Minimum	3.3	2.7	0.4	21.4	-21.6	2.1	1.8	3.8	42.0	-4.3	5.0	3.5	7.2	9.7	32.6
		Maximum	10.1	9.2	58.5	79.1	49.6	13.1	7.1	15.2	81.9	59.3	22.2	10.5	27.6	85.0	58.1
		N	7	7	7	7	7	7	7	7	7	7	7	7	7	7	7
**Combination therapy (ITZ + KI)**	**cure**	Median	10.8	5.4	9.0	70.0	36.9	7.9	5.4	16.1	69.5	36.5	8.1	4.7	13.0	59.3	39.9
		Minimum	3.9	2.9	0.1	36.0	-63.2	3.9	2.4	2.6	1.3	7.3	3.6	2.6	5.9	40.5	-3.1
		Maximum	24.5	10.1	63.2	91.9	71.5	12.1	7.4	38.3	90.7	61.5	18.8	12.2	33.3	85.4	66.0
		N	16	15	16	16	16	16	16	16	16	16	16	15	16	16	16
	**failure**	Median	5.6	6.7	11.0	65.0	24.0	7.6	4.6	8.6	42.0	19.1	7.2	6.3	11.0	54.2	46.9
		Minimum	3.7	3.7	10.2	47.4	6.2	6.1	3.6	5.2	29.6	10.9	6.7	4.1	10.0	25.6	26.6
		Maximum	7.4	9.6	11.8	82.6	41.8	9.2	5.6	12.0	54.4	27.3	7.8	8.4	12.1	82.9	67.3
		N	2	2	2	2	2	2	2	2	2	2	2	2	2	2	2

SI-Zym: Stimulation index of Zymosan stimulated cells; SI-Sp: Stimulation index of *Sporothrix* stimulated cells; %RHD: percentage of basal activation; %Zym: Percentage of Zymosan stimulated cells; %Sp: Percentage of *Sporothrix* stimulated cells

ITZ: Itraconazole; KI: Potassium iodide

T1: before the beginning of the treatment); T2: 1-2 months into the treatment); T3: outcome – clinical cure or treatment failure.

*Negative values indicate that the background percentage was higher than the percentage of stimulated cells
